# Case Report: Systemic Treatment and Serial Genomic Sequencing of Metastatic Prostate Adenocarcinoma Progressing to Small Cell Carcinoma

**DOI:** 10.3389/fonc.2021.732071

**Published:** 2021-09-27

**Authors:** XiaoJun Lu, Wenwen Gao, Yu Zhang, Tao Wang, Hongliang Gao, Qing Chen, Xiaolei Shi, Bijun Lian, Wenhui Zhang, Xu Gao, Jing Li

**Affiliations:** ^1^ Department of Urology, Shanghai Changhai Hospital, Second Military Medical University, Shanghai, China; ^2^ Department of Oncology, Shidong Hospital, Affiliated to University of Shanghai for Science and Technology, Shanghai, China; ^3^ Department of Bioinformatics, Center for Translational Medicine, Second Military Medical University, Shanghai, China; ^4^ Department of Urology, The First Affiliated Hospital of Zhengzhou University, Zhengzhou, China; ^5^ Department of Urology, The 903th PLA Hospital, Hangzhou, China

**Keywords:** neuroendocrine differentiation, small cell carcinoma, whole-exome sequencing, neuroendocrine prostate cancer, clonal evolution

## Abstract

Small cell carcinoma (SCC)/neuroendocrine prostate cancer (NEPC) is a rare and highly aggressive subtype of prostate cancer associated with an AR(androgen receptor)-null phenotype and visceral metastases. This study presents a 44-year-old man originally diagnosed with metastatic hormone-sensitive prostatic adenocarcinoma. After 6-month androgen deprivation therapy (ADT) combined with docetaxel, the patient developed paraplegia. Laminectomy was performed, and a thoracic vertebral biopsy revealed neuroendocrine differentiation and mixed adenocarcinoma. The patient developed liver metastases and experienced stable disease for 4 months following etoposide combined with cisplatin and pembrolizumab. Seminal vesicle biopsy after chemotherapy revealed small-cell cancer. The prostate biopsy specimen also indicated pure SCC. We witnessed the dynamic evolution from pure adenocarcinoma to fully differentiated SCC, leading to obstruction and death. In addition, whole-exome sequencing was performed on both biopsy specimens of the thoracic vertebra at the beginning of castration resistance and that of seminal vesicle after multiple lines of treatment failure. Utilizing phylogenetic reconstruction, we observed that both samples shared a common ancestor clone harboring aberrations in the *TP53*, *RB1*, and *NF2* genes. We also discovered that driver events in the private subclones of both samples, such as alterations in *CDC27* and *RUNX1*, might have played a significant role in tumor progression or even neuroendocrine differentiation. Tumor biopsy and IHC assessment must be repeated at different stages of progression, because of intrapatient spatial and temporal heterogeneity of adenocarcinoma *versus* SCC/NEPC. Although, typical treatments including ADT, docetaxel, etoposide, cisplatin, and pembrolizumab provided temporary response, the patient still had a poor prognosis.

## Introduction

The androgen receptor (AR) regulates of growth and proliferation of prostate cancer (1). Androgen-deprivation or highly potent AR-targeted therapies, such as enzalutamide, remain the mainstay for the systematic treatment of metastatic hormone-sensitive prostate cancer (mHSPC). However, majority of the mHSPC cases eventually develop to metastatic castration-resistant prostate cancer (mCRPC) after long-term androgen deprivation therapy (ADT). An important mechanism in treatment-resistant prostate cancer development might be associated with neuroendocrine differentiation. Neuroendocrine prostate cancer (NEPC) is an aggressive variant of prostate cancer, characterized by pure or mixed neuroendocrine differentiation. Histologically, small cell neuroendocrine carcinoma is among the highest-grade and poorly differentiated neuroendocrine tumors. Small cell carcinoma constitutes 0.5–2% of prostate cancer cases, whereas 10–20% of CRPC cases; however, the current prevalence of SCC/NEPC after intense therapeutic pressures designed to inhibit AR signaling might be higher (2). Using molecular classifiers some studies have supported the clonal evolution trajectory of NEPC from adenocarcinoma (3).

Here, we present a case that shows the transformation process from adenocarcinoma to neuroendocrine cells and small cell prostate cancer at a very late stage. We found the coexistence of heterogeneous subtypes in the whole body during treatment, which elicited different reactions to different treatments. Furthermore, we performed serial molecular profiling and explored the clonal evolution pattern of NEPC to small cell carcinoma in this patient after treatment to help understand the potential mechanism of this evolutionary path.

## Case Description

**Figure 1:**
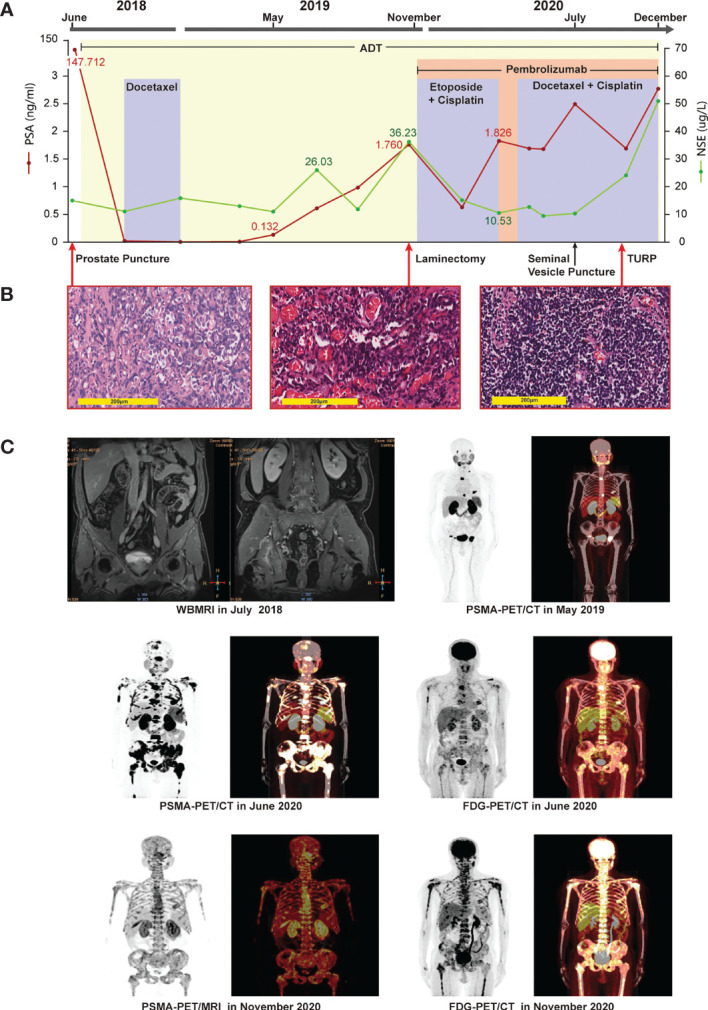
Clinical timeline, systemic therapy, imaging data, and pathologic diagnosis. **(A)** systemic therapy: continuous adjusted treatment protocols in disease progression and changes in the levels of PSA and NSE during the treatment course. **(B)** pathologic diagnosis: (left picture) HE staining revealing prostate adenocarcinomas in the prostate puncture tissue; (middle picture) HE staining revealing metastatic prostate adenocarcinoma with neuroendocrine differentiation in the thoracic vertebral tissue; (right picture) HE staining revealing small-cell cancer in the tissue after TURP surgery. Magnification: ×200. **(C)** Imaging data: whole-body magnetic imaging resonance showing changes in the prostate lesion before treatment in July 2018. PSMA-PET/CT showing multiple bone lesions in May 2019, after termination of docetaxel chemotherapy, and elevation of the levels of the patient’s PSA to 0.132 ng/mL. PSMA-PET/CT in June 2020 showing metastases in the left seminal vesicle, liver, and multiple bones. FDG-PET/CT in June 2020 showing metastases in the left seminal vesicle, liver, and multiple bones. PSMA-PET/MRI in November 2020 showing a metastasis in the posterior wall of the bladder, diffuse bone metastases, bilateral pleural effusion liquid, and a chronic subdural hematoma in bilateral brain. FDG-PET/CT in November 2020 showing multiple metastases in the bones and liver, and bilateral pleural effusion liquid, but no abnormalities in brain.

In June 2018, a 44-year-old man was originally diagnosed with prostate cancer (PCa) confirmed using prostate biopsy, which indicated a Gleason score of 4 + 5 = 9 prostate adenocarcinomas involving all 15 cores (**Figure 1A**). From June 2018 to December 2020, the patient presented with three stages of prostate cancer, according to treatment, mHSPC, mCRPC, and small cell. Immunohistochemical (IHC) assessment demonstrated tumors to be positive for prostate-specific membrane antigen (PSMA), P504s, and P501s (**Table 1**). Prostate-specific antigen (PSA) levels were initially elevated at 147.7 ng/mL. Whole-body magnetic resonance imaging (WB-MRI) indicated metastases involving the prostate capsule, bilateral seminal vesicles, pelvic lymph nodes, and extensive osteosclerotic lesions (**Figure 1C**). Hence, the patient was diagnosed with cT3bN1M1b stage cancer. Because of the high tumor burden, we began treatment for mHSPC with ADT plus docetaxel at 75 mg/m^2^ for six cycles. Following this, the PSA levels showed a rapid and significant improvement, dropping to 0.004 ng/mL in December 2018. Both WB-MRI and a bone scan showed no obvious active lesions in the prostate, pelvic lymph nodes, and bones (**Supplementary Figure 1**).

**Table 1:** Immunohistochemical (IHC) assessment of puncture tissue of prostate, thoracic vertebral biopsy specimen, seminal vesicle tissue, and prostate after transurethral resection of prostate (TURP).

IHC Assessment	mHSPC	mCRPC	Small Cell	Small Cell
	Puncture Tissue	Thoracic Vertebra	Seminal Vesicle	Prostate Tissue
NKX3.1	(+)	(+)	(-)	(-)
PSMA	(+)	(+)		(-)
ERG	(-)	(-)		(+)
AR		80%	30%	
CgA		(+)	(+)	(+)
CD56		(+)	(+)	(+)
Syn		(+)	(+)	(+)
NSE		(-)		
CK20		(-)		
P504s	(+)	(+)		(+)
P501s	(+)	(+)	(-)	/
CAM5.2		(+)	(+)	(+)
Pten				(+)
PD-1		(-)		
PDL-1		(-)		
KI-67	40%	40%	100%	100%

(+) represents positive assessment. (-) represents negative assessment. Blank indicates no protein staining.

Although ADT plus docetaxel therapy achieved periodic results in the treatment of mHSPC, his levels of PSA increased continuously with ADT therapy alone over 5 months after stopping chemotherapy, reaching 0.132 ng/mL in May 2019. Meanwhile, PSMA positron emission tomography (PET)/computed tomography (CT) revealed the presence of metabolically active lesions in multiple bones, but not in the prostate (**Figure 1C**). Therefore, we assumed that the mHSPC had progressed to mCRPC. In July 2019, we treated the patient with ADT and abiraterone for over 3 months. However, his levels of PSA continued to increase, and a severe complication of paraplegia occurred in October 2019. The patient underwent laminectomy in November 2019, which led to improved motor capacity. The posttreatment thoracic vertebral biopsy specimen revealed metastatic prostate adenocarcinoma with neuroendocrine differentiation (**Figure 1B**). IHC assessment demonstrated tumors to be positive for PSMA, P504s, NKX3.1, synaptophysin (Syn), CD56, chromogranin A (CgA), AR(+, 80%), and KI67(+, 40%) (**Table 1**). A fresh portion of the vertebral specimen was sent for tumor sequencing.

**Figure 2:**
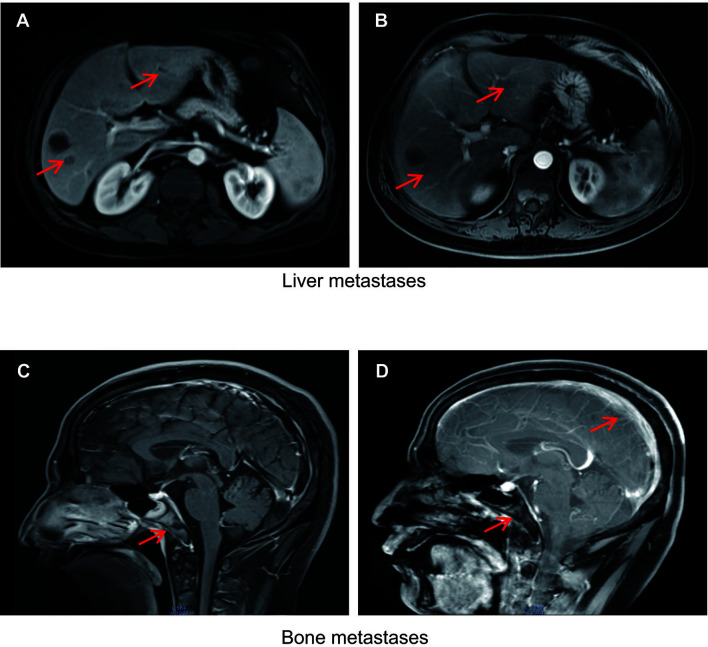
**(A)** Liver metastases detected using magnetic resonance imaging (MRI) in November 2019. **(B)** Response of liver metastases detected using MRI in January 2020 after treatment with etoposide combined with cisplatin and continued ADT plus pembrolizumab. Metastases are indicated by red arrows. **(C)** Clivus and adjacent right sphenoid bone metastases detected by MRI in April 2020. **(D)** Meningeal clivus and adjacent right sphenoid metastases in November 2020. Metastases are indicated by red arrows.

One month later, the patient had elevated levels of transaminases. Magnetic resonance imaging (MRI) revealed new lesions in the liver (**Figure 2A**). Neuron-specific enolase (NSE) levels were elevated at 36.23 ng/mL (**Figure 1A**). Based on the pathology and clinical features, we initiated etoposide at 80 mg/m^2^ combined with cisplatin at 25 mg/m^2^, and continued ADT therapy plus pembrolizumab specific for NEPC. After two cycles, an MRI showed that the liver lesions had almost disappeared (**Figure 2B**).

Following four cycles of etoposide combined with cisplatin plus pembrolizumab, the patient experienced severe general bone pain and blurred vision. His levels of PSA were transiently decreased, but increased again to 1.826 ng/mL in the last four cycles, whereas his levels of NSE decreased to 10.53 ng/mL. A new MRI revealed metastases involving the clivus and adjacent right sphenoid bone (**Figure 2C**). Hence, we hypothesized the coexistence of mixed adenocarcinoma and neuroendocrine components. In particular, we considered that the NSE signal showed the efficiency, while the levels of PSA represented the growth of adenocarcinoma components. Therefore, we attempted treatment with docetaxel at 75 mg/m^2^, cisplatin at 25 mg/m^2^, and continued ADT therapy plus pembrolizumab. Notably, the patient recovered his vision and experienced pain relief. After four cycles, his symptoms improved significantly. However, PSMA-PET/CT revealed metabolically active lesions on the left seminal vesicle, liver, and multiple bones, indicating cancer progression (**Figure 1C**). In addition, a seminal vesicle biopsy in July 2020 revealed small cell carcinoma. IHC assessment demonstrated tumors to be positive for CAM5.2, Syn, CD56, CgA, AR (+, 30%), and Ki-67(+, 100%), but negative for NKX3.1 and P501s (**Table 1**). A fresh portion of this seminal vesicle specimen was sent for tumor sequencing. Three months later, the patient underwent palliative transurethral resection of prostate (TURP) for dysuria. The prostate biopsy specimen indicated small cell carcinoma. IHC assessment demonstrated tumors to be positive for P504s, CAM5.2, Syn, CD56, CgA, ERG, PTEN, and Ki-67(+, 100%), but negative for NKX3.1 and PSMA.

## Molecular Tumor Board

### Pathology and Molecular Evolution From Adenocarcinoma to NEPC to SCC

Neuroendocrine differentiation in adenocarcinoma should be detected in a timely manner. Both the elevation of serum NSE levels and the presence of visceral metastases indicate the development of neuroendocrine differentiation in prostate cancer. Despite their high sensitivity, the CgA and NSE serum neuroendocrine markers lack specificity for SCC/NEPC (2, 4, 5). Pathological diagnosis is the most reliable diagnosis, and the acquisition of reliable biopsy specimens usually depends on good compliance of the patient and the puncture technique of the doctor (6). We obtained four pre- and posttreatment biopsy specimens, which revealed the histological progression of tumors (**Table 1**) (**Supplementary Figure 2**). PSMA, P504s, and NKX3.1 were expressed in nearly all prostatic adenocarcinomas. Only these markers were expressed at initial diagnosis, indicating the adenocarcinoma origin of the tumor. The CgA, Syn, and CD56 markers are expressed in nearly all prostate cancers with neuroendocrine differentiation (7). Following its progression to mCRPC, IHC assessment of the thoracic vertebral biopsy specimen revealed positive signals for PSMA, P504s, NKX3.1, Syn, CD56, and CgA, which indicated adenocarcinoma admixed with NEPC after intense therapeutic pressures of ADT therapy. Likewise, IHC assessment of the seminal vesicle biopsy specimen showed positive signals for CAM5.2, Syn, CD56, and CgA, but negative for NKX3.1 and P501s, indicating the predominance of SCC/NEPC in the patient during this period. Under the pressure of treatment, NEPC transformed into a poorly differentiated small cell phenotype, losing the expression of adenocarcinoma origin-specific markers, such as PSMA and NKX3.1.

### Functional and Clinical Significance of Specific Mutations in This Case

As the special genetic pattern of NEPC reflects its cellular origin, the genetic alterations in two samples collected at different stages were investigated using whole-exome sequencing. The first was a mixed adenocarcinoma and neuroendocrine cancer sample (M-sample) at CRPC, whereas the second was a pure SCC/NEPC (S-sample) at the last stage. We described the mutational landscape and applied phylogenetic reconstruction to analyze the dynamic clonal progression. We estimated and clustered the cancer cell fraction (CCF) to track and visualize the clonal evolution of each tumor using PyClone (8).

**Figure 3:**
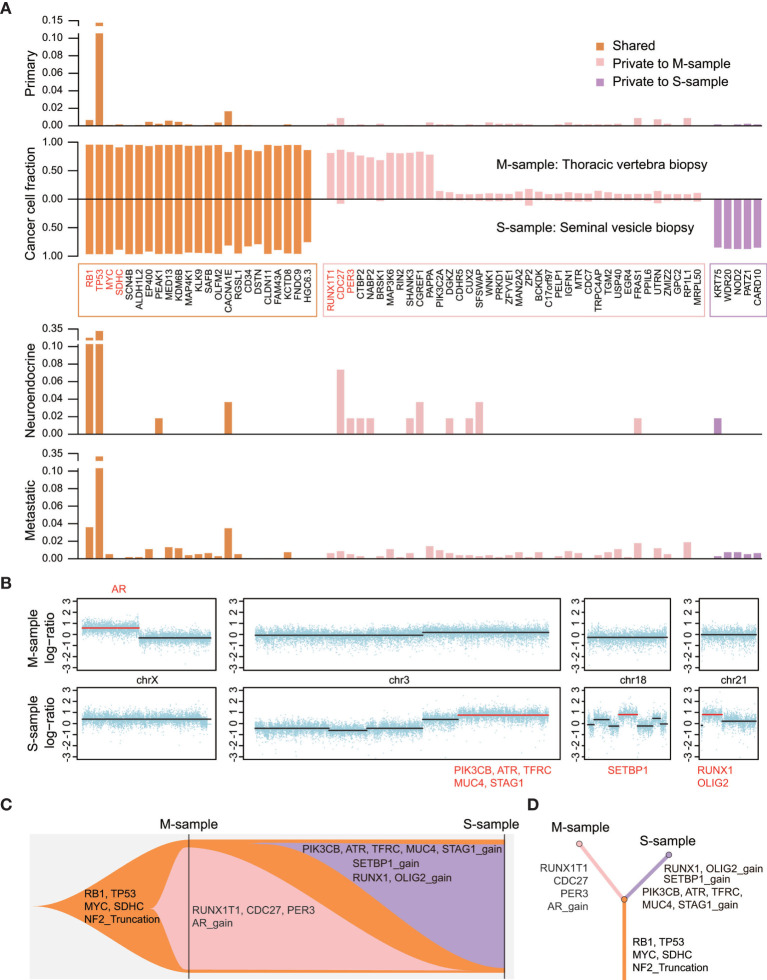
The altered landscape and phylogenetic reconstruction of the two samples. **(A)** The second panel shows the cancer cell fraction estimated by using PyClone and calculated using the read depth of mutations, copy numbers, and purity of tumors. Other panels are showing the frequency of mutations in three types of prostate cancer using public data from the cBioPortal (1400 primary tumors, 54 NEPC samples, and 880 mCRPC samples). Important functional cancer genes are marked in red. **(B)** Overview of copy number alterations and cancer genes encompassed in segments are shown in red. **(C, D)** Fishplot indicating the dynamic clonal progression of the tumor, and clonal evolution tree showing the phylogenetic relationship between the two samples.

We observed that both samples shared the most recent common ancestor (MRCA) encompassing 23 mutations and an exon1-exon13 truncation of the neurofibromin 2 (*NF2*) gene, indicating their common origin (**Figure 3**). Notably, a double deletion of *TP53* and *RB1* was identified at the initiation of neuroendocrine stages, while a truncation of *NF2* resulted in sensitivity to platinum chemotherapy (9). A specific subclone harboring 37 mutated genes and an AR amplification was observed in the M-sample. We integrated the mutation frequency of all mutated genes in different types of prostate cancer in this patient, according to the existing data using “cBioPortal” (http://www.cbioportal.org) as previously described (10) (**Figure 3A**). We observed that the *CDC27* oncogene was frequently mutated in NEPC samples (7.4%, 4/54 public samples). *CDC27* has been recognized as either a tumor suppressor gene or an oncogene in different neoplasms. Furthermore, *CDC27* increases the stemness of cancer stem cells in colorectal cancer (11). A private subclone harboring three copy number alterations and five mutations was detected in the S-sample. Among them, the *RUNX1* gene, a transcription factor playing key roles in the regulation of stem cell fate, might be driving the neuroendocrine progression of tumor (12). Although Beltran et al. observed that the *RUNX1* copy number deletion was meaningful in NEPC, a *RUNX1* amplification could also occur, further supporting the key role of *RUNX1* in neuroendocrine-differentiated tumors (13). Consequently, the two samples obtained individual subclones after MRCA in the clonal evolutionary tree (**Figures 3C, D**). Some driving events in these subclones might have played a key role in tumor progression or even in promoting neuroendocrine differentiation.

## Discussion

### Potential Strategies to Target the Pathway and Implications for Clinical Practice

Precision oncology is based on tumor biopsies and sequencing to identify therapeutic targets. Here, two posttreatment biopsy specimens with histologic progression were analyzed using whole-exome sequencing. An AR amplification was detected in the first mixed tumor sample but not in the second pure SCC/NEPC sample, indicating the downregulation of AR in NEPC compared with that in adenocarcinoma (5, 13). In addition, the inactivation of *RB1* and *TP53* tumor suppressor genes is frequently associated with small cell cancer (14, 15), and is a key mechanism of resistance to antiandrogen therapy and lineage plasticity (16, 17). Identical mutations in the DNA-binding domains of TP53, RB1, and MYC have also been observed in both NGS profiles, indicating the possible derivation of NEPC from adenocarcinoma (18–20). We attempted to identify therapeutic targets, such as DNA damage repair (DDR) pathway genes (*BRCA1*, *BRCA2*, *ATM*, and *CDK12*), which could be paired with poly ADP-ribose polymerase (PARP) inhibitors (6, 21). However, we did not detect any alterations in DDR pathway genes in the NGS profiling. Similarly, other studies have also shown the rare occurrence of alterations in DDR pathway genes in SCC/NEPC (2). We observed alterations in *PIK3CA* and *NF2*, which could be associated with the phosphatidylinositol-3-kinase/mammalian target of rapamycin (PI3K/mTOR) pathway. However, the curative effect of mTOR inhibitors in prostate cancer remains uncertain (22, 23). Considering the uncertain curative effect and the poor physical condition of the patient, we elected the regimen recommended by the guidelines (6, 24).

Prostate cancer with neuroendocrine differentiation shows remarkable clinical heterogeneity, as revealed by the constantly changing clinical symptoms, such as bone pain, liver metastases, and meningeal metastases. We deduced that adenocarcinoma and SCC/NEPC alternately dominated the shift in the process of disease. We elected ADT combined with docetaxel as the initial treatment, but changed to etoposide combined with cisplatin and pembrolizumab after the detection of neuroendocrine differentiation. In this case, the key advantage of the approach used is the timely correction of treatment strategy according to the pathological type and the construction of tumor subclone structures using sequential sampling. In addition, we advanced chemotherapy at the initial diagnosis based on the NCCN guidelines (6), and timely chose the regimen with etoposide and platinum (EP) + PD1 when the tumor was revealed to be neuroendocrine differentiation and mixed adenocarcinoma. Despite accomplishing a temporary response, regrettably, we could not find a regimen that could ideally control both adenocarcinoma and SCC/NEPC to improve the prognosis. We have also supplemented our report with the research on the origin of SCC/NEPC from the perspective of clonal evolution. Previous studies have reported the transdifferentiation phenomenon of NEPC and its molecular changes, that is, prostate adenocarcinoma cells could transform into a neuroendocrine phenotype (25, 26). We described here the clonal evolution from mixed adenocarcinoma and neuroendocrine cancer to pure SCC/NEPC. Meanwhile, some molecular alterations have also been provided, which could hopefully be used to monitor the progress of SCC/NEPC in the future.

Notably, this case study has some limitations. While longitudinal sampling combined with bulk sequencing could distinguish tumor subclones and monitor tumor progression, for the special tumor type observed in this case—with higher heterogeneity and more complex cellular components—single-cell sequencing may be a better and more accurate solution (27). Alternatively, in a previous report, transcription factors have been utilized for the molecular subtyping of small cell lung cancer; however, this has not been applied in clinical practice (28). Accordingly, further research is required to explore the value of transcription factor typing to guide clinical treatment.

### Patient Update

Despite a periodic clinical response, the patient died from respiratory failure, severe anemia, infection, and brain edema in December 2020. The last MRI showed metastases of the meninges, clivus, skull, and sphenoid bone (**Figure 2D**).

### Key Points

If the IHC assessment of the initial puncture biopsy tissue detects potential neuroendocrine differentiation, doctors should offer patients more frequent imaging examination and analyses of serum markers.

Tumor biopsy and IHC assessment must be repeated at different stages of the disease to judge the progress of neuroendocrine differentiation and adjust the treatment regimen.

Tumor sequencing remains necessary even though changes might not be necessarily detected, as it could help clinicians better understand the genomic changes during neuroendocrine differentiation.

## Data Availability Statement

The original contributions presented in the study are included in the article/**Supplementary Material**. Further inquiries can be directed to the corresponding authors.

## Author Contributions

Conception/design: JL and WZ. Provision of study material or patients: TW and HG. Collection and/or assembly of data: YZ and XS. Data analysis and interpretation: WZ and QC. Manuscript writing: XL and WG. Manuscript revision: XG. Final approval of manuscript: BL and JL. All authors contributed to the article and approved the submitted version.

## Funding

This study was supported by the Shanghai Rising-Star Program (20QA1411800) and National Natural Science Foundation of China (82022055).

## Conflict of Interest

The authors declare that the research was conducted in the absence of any commercial or financial relationships that could be construed as a potential conflict of interest.

## Publisher’s Note

All claims expressed in this article are solely those of the authors and do not necessarily represent those of their affiliated organizations, or those of the publisher, the editors and the reviewers. Any product that may be evaluated in this article, or claim that may be made by its manufacturer, is not guaranteed or endorsed by the publisher.

## Supplementary Material

Supplementary Figure 1 Figure WB-MRI shows there were no obvious active lesions in prostate, pelvis lymph nodes, and bones all over the body in January 2019 after six chemotherapy cycles of docetaxel along with ADT therapy.

Supplementary Figure 2 Positive IHC assessment of puncture tissue of prostate, thoracic vertebra biopsy specimen, seminal vesicle tissue, and prostate after TURP.

Supplementary Table 1 Summary whole exome sequencing findings from the thoracic vertebra biopsy specimen and the seminal vesicle specimen. Variants were identified including single nucleotide variants(SNV), small insertion-deletions, and copy number alterations. The cancer cell fractions(CCF) and clusters of each mutations are also indicated.
