# Supplementary figures and images for: Case Report: Systemic Treatment and Serial Genomic Sequencing of Metastatic Prostate Adenocarcinoma Progressing to Small Cell Carcinoma
Source: Front Oncol. 2021 Sep 27;11:732071. doi: 10.3389/fonc.2021.732071 (PMC8503647; doi:10.3389/fonc.2021.732071)

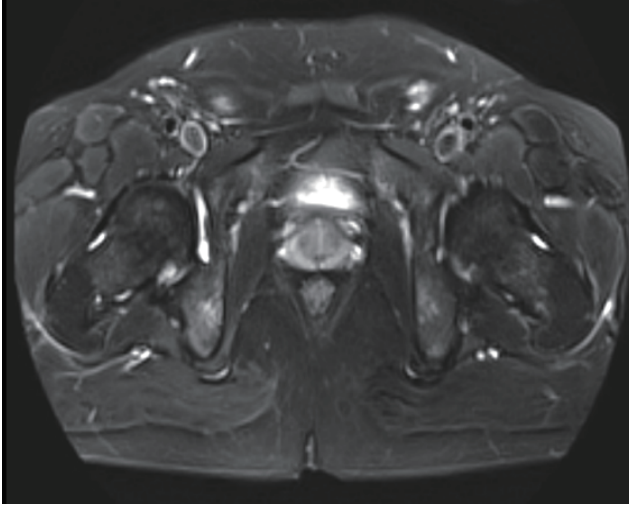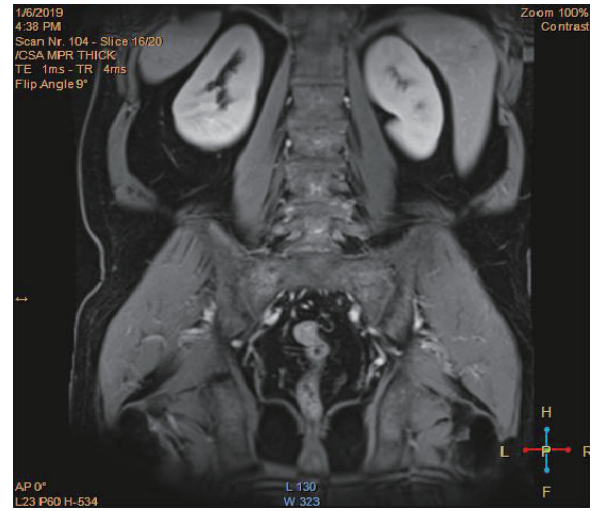

Supplement: Supplementary Figure 1 — Figure WB-MRI shows there were no obvious active lesions in prostate, pelvis lymph nodes, and bones all over the body in January 2019 after six chemotherapy cycles of docetaxel along with ADT therapy. [file Image_1.pdf]

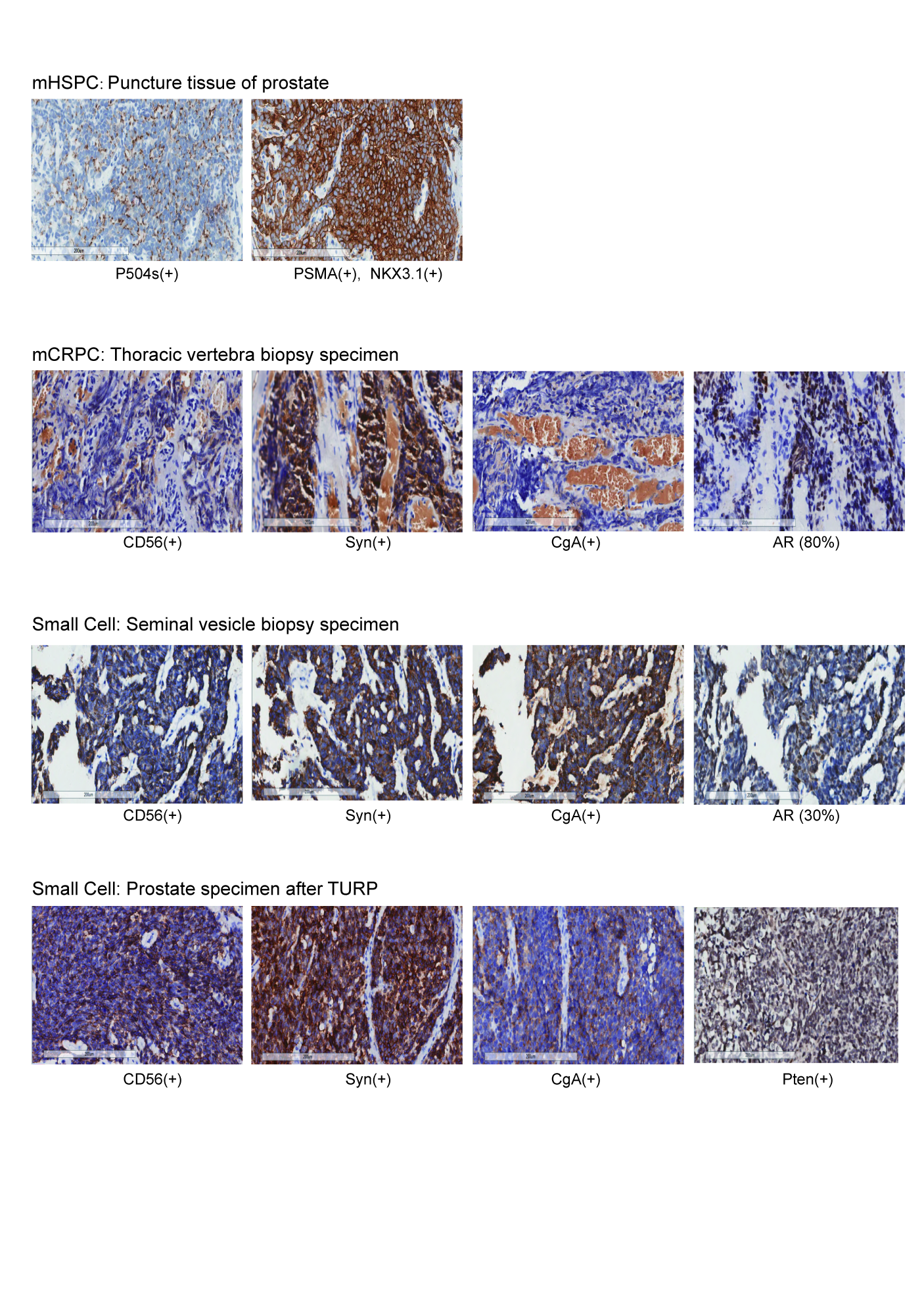

Supplement: Supplementary Figure 2 — Positive IHC assessment of puncture tissue of prostate, thoracic vertebra biopsy specimen, seminal vesicle tissue, and prostate after TURP. [file Image_2.tif]
